# Under the Radar: Immune Evasion, Pathogenesis and Control of HIV Infection

**DOI:** 10.3390/ijms262311381

**Published:** 2025-11-25

**Authors:** Ferran Tarrés-Freixas, Benjamin Trinité, Jorge Carrillo, Julià Blanco

**Affiliations:** 1 IrsiCaixa, Ctra del Canyet S/N., 08916 Badalona, Spain; btrinite@irsicaixa.es (B.T.); jcarrillo@irsicaixa.es (J.C.); 2 CIBERINFEC, 28029 Madrid, Spain; 3 Germans Trias i Pujol Research Institute (IGTP), 08916 Badalona, Spain; 4 Faculty of Medicine, University of Vic-Central University of Vic (UVic-UCC), 08500 Vic, Spain

**Keywords:** viral pathogenesis, immune responses, antiretrovirals, vaccines, gender

## Abstract

The human immunodeficiency virus (HIV) is a retrovirus discovered in 1983 as the causative agent of acquired immunodeficiency syndrome (AIDS). Following several zoonotic spillover events from non-human primates, the virus spread between humans for more than 60 years under the radar. HIV infects and kills CD4 T cells, the cells that coordinate adaptive immune responses. Primoinfection is associated with a flu-like symptomatology and chronic infection is clinically silent, and mostly not diagnosed, contributing to viral spread and leading to fatal long-term outcomes. HIV genome codes for a poor reading-proof reverse transcriptase, which facilitates high sequence variability, particularly in the envelope glycoprotein complex, the sole external viral protein and main target of humoral immune responses. This antigenic variability precludes the development of an efficacious vaccine despite 40 years of research. In contrast, the development of antiretroviral drugs represents a scientific and medical success which saved the lives of millions of infected people and provides today an excellent protection against AIDS, although it does not permit viral eradication. Indeed, HIV can integrate its genome in target cells and generates a pool of latently infected cells which escape eradication by both the natural immune response and treatments. In summary, the efforts to tackle HIV have been suboptimal, and the virus has infected more than 90 million people and caused 44 million deaths worldwide. In the absence of a vaccine, a better deployment of available preventative and therapeutic tools is needed, particularly in geographical areas and communities with the highest incidence of infection.

## 1. A Brief History of HIV. From SIV to HIV

Human Immunodeficiency Virus (HIV), the etiologic agent of acquired immunodeficiency syndrome (AIDS), has a relatively recent history but has had a profound impact worldwide over the last 40 years. In 1981, the first cases of AIDS were identified in the United States [1], and two years later, the virus was discovered as the causative agent of AIDS by Françoise Barré–Sinoussi and Luc Montagnier at the Pasteur Institute [2] in a controversial race with Robert Gallo [3]. Since then, it is estimated that 91.4 million people have been infected by HIV, and 44.1 million people have died due to AIDS-related events [4]. However, HIV has an older, albeit silent, history during which it established the pillars that it would eventually fuel the current pandemic.

HIV birth can be traced back to multiple simian immunodeficiency viruses (SIV) zoonotic infections in humans. SIV infection in humans is not rare in areas where humans and non-human primates (NHP) cohabitate. It is estimated that 2.3% of the population in Cameroon has been exposed to SIV, and this prevalence rises to 7.8–17.1% in rural villages where poachers hunt NHP for bushmeat [5]. Therefore, several zoonotic transmissions of SIV to humans have been reported, contributing to the current spread of HIV in humans.

**Figure 1:**
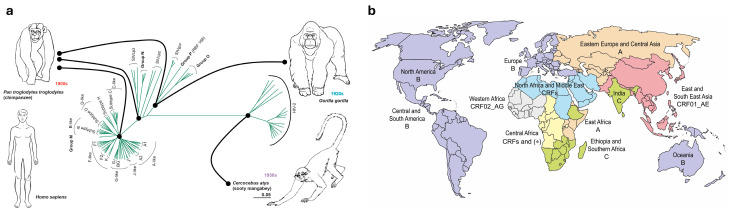
(**a**) Phylogenetic tree of HIV-1 groups M, N, O and P, HIV-2 and SIV from chimpanzee (SIVcpz), gorilla (SIVgor) and sooty mangabey (SIVsmm). HIV-1 group M, the main driver of the worldwide pandemic, is further divided into its clades or subtypes. The sequence divergence scale is indicated. Connectors indicate main transmission events. Adapted from Tebit and Arts, 2011 [17]. (**b**) Current global distribution of HIV-1 group M clades. Colouring represents the predominant HIV-1 clade(s) in each geographic area (A, B, C, or CRFs). CRF: circulating recombinant form. (*) indicates a high heterogeneity with no prevalent HIV-1 clade.

Nowadays, HIV is a highly diverse virus. It has been classified into different types (HIV-1 and HIV-2) and groups; these groups are M, N, O and P from HIV-1 and groups A-I from HIV-2 [6]. Phylogenetic studies demonstrate that each one of these groups represents a single and different cross-species transmission from NHP to human [7], meaning that there have been at least 13 known individual events in which SIV managed to cross the species barrier (Figure 1a) [8]. For instance, HIV-1 group M (“M” stands for “major”) and group N most resemble chimpanzees’ SIV (SIVcpz) [9], while HIV-1 groups O and P derive from SIV found in gorillas (SIVgor) [10]. Additionally, the HIV-2 virus, which is endemic in West Africa and less pathogenic than HIV-1, is closely related to sooty mangabeys SIV (SIVsmm) [11].

While HIV types and groups emerged from different cross-species transmissions, HIV subtypes or clades within a group were derived from the diversification of a common ancestor. In this regard, HIV-1 group M accounts for more than 90% of HIV infections worldwide and will be the focus of this review.

The pandemic HIV-1 group M originated in the early 20th century, likely in rural areas of southeast Cameroon. The virus then travelled in its host to Kinshasa in the Democratic Republic of Congo (DRC) through the Congo River, where it settled as an epidemic agent [12]. Kinshasa is believed to be the focus of HIV-1 group M, since it houses the highest variability of this HIV group [13]. Evolutionary studies estimate that the most recent common ancestor (MRCA) of group M time of is around 1920 [14]. In fact, the two oldest HIV samples conserved, ZR59 and DRC60 (subtypes D and A, respectively), were retrospectively obtained in Kinshasa. For 40 years, HIV-1 group M remained as an unidentified epidemic agent circulating in Kinshasa and its neighbouring cities, where it diversified into multiple clades (namely subtypes A, B, C, D, F, G, H, J and K). Migration movements and changes in post-independence DRC helped HIV-1 group M to grow exponentially and expand worldwide. Estimates indicate that HIV-1 arrived in Haiti around 1964, when professional workers returned from DRC after helping to fill administrative gaps caused by DRCs independence from Belgium, and from there the virus entered the United States of America (USA) through migratory movements [15].

By 1981, the Centres for Disease Control had reported in certain populations a series of unusual diseases such as Pneumocystis carinii pneumonia, Kaposi’s sarcoma and opportunistic infections that were associated with cell-mediated immune deficiencies and in 1982, the term AIDS was coined [16]. By that time, HIV was present worldwide, and an AIDS diagnosis was a death sentence, but the discovery of HIV as the causative agent of AIDS in 1983 paved the way towards the development of antiretroviral therapies to tackle the virus [2].

Currently, HIV clades are globally distributed, but their prevalence differs across the distribution areas (Figure 1b). Subtype B is the predominant strain in Western Europe, America and Australia, clade C is the predominant clade in Southern Africa, CRF01_AE subtype dominates in Southeast Asia, while central Africa heterogeneously houses most variants, circumventing HIV-1 origin (Figure 1b) [8]. Genetic diversity of HIV-1 group M within a clade represents up to 15% of variability, while it can reach up to 40% variability between clades [16].

## 2. Molecular Events in the Life Cycle of HIV

### 2.1. Viral Genome and Structure

Understanding the capacity of HIV to silently spread worldwide requires a close look at the molecular events occurring during viral replication. These events are defined by the ability of viral proteins to hijack cellular functions for their own benefit and to block antiviral host cell responses. This, in turn, will determine the outcome of the infection process and will delineate the individual immune response and silent viral pathogenesis, which will further impact viral transmission and epidemiology of the pandemic.

**Figure 2:**
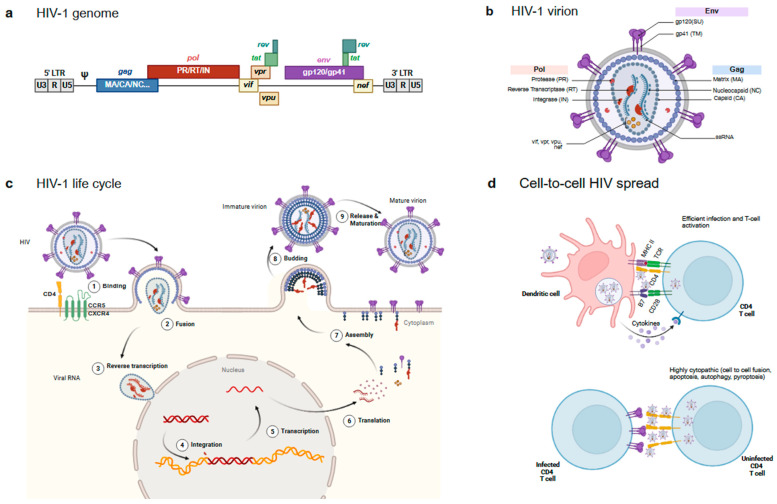
Main HIV features. (**a**) Structure of the HIV genome. Main HIV genes (*gag*, *pol* and *env*) are depicted in blue, red and purple, respectively, and indicate the main resulting proteins after proteolytic processing. Accessory proteins are depicted in yellow/green. (**b**) Structure of the mature HIV particle. The figure shows the main proteins coloured according to panel a: Env on the surface of the particle, the different gag products organising inner particle structures, and pol and accessory proteins packaged into the viral particle. (**c**) HIV-1 life cycle. The figure shows the main steps of the virus infection. See text for details. (**d**) Cell-to-cell virus transmission events. The two main cell-to-cell contacts involved in HIV spread are shown. An infectious synapse between an HIV loaded DC and a CD4 T cell (**Top**) and a virological synapse between an infected and an uninfected CD4 T cell (**Bottom**).

HIV-1 belongs to the lentiviruses subclass of the *Retroviridae* family. Similarly to all retroviruses, its genome, which is roughly 10 Kb long, contains two long terminal repeat (LTR) sequences in the 5′ and the 3′ ends and the main genes defining simple retroviruses, namely the group-specific antigen gene (*gag*) coding for structural proteins (matrix/MA, capsid/CA and nucleocapsid/NC and other small proteins); the polymerase gene (*pol*) coding for all the viral enzymes (protease/PR, reverse transcriptase/RT and integrase/IN); and the envelope gene (*env*) coding for the external envelope glycoprotein complex (Env) of the virus. The HIV genome also codes for different accessory proteins, which are a feature of complex retroviruses (Figure 2a). These relatively small proteins (tat, vif, vpr, vpu, rev and nef) are responsible for a plethora of functions that have a major contribution to the unique HIV replication life cycle.

The HIV-1 mature viral particle shows a spherical shape with a lipid bilayer exposing the Env protein and containing the viral structural proteins, the matrix protein associated with the inner layer of the membrane, the capsid protein arranged in a truncated cone structure concealing the viral enzymes and two single-stranded ribonucleic acid (ssRNA) molecules coding for the full HIV-1 genome (Figure 2b).

### 2.2. The Viral Life Cycle

The viral life cycle starts when HIV-1 Env displayed on the surface of the virus engages the CD4 receptor on target cells [18]. Env is a complex and plastic protein generated by the trimerisation of three gp160 molecules. Each gp160 monomer is cleaved by the cellular protease furin to generate two different subunits (gp120 or SU for surface and gp41 or TM for transmembrane) joined by non-covalent interactions. Gp120 acts as the receptor-recognising subunit, while gp41 harbours the fusion machinery [19]. The CD4 binding site (CD4bs) on gp120 recognises CD4 and initiates a sequence of conformational changes that allows Env to bind a coreceptor, either of the chemokine receptors CCR5 or CXCR4 [20,21]. The coreceptor that each strain of HIV-1 binds to defines the tropism that each virus has (R5, X4R5 or X4). Upon binding to the receptor and coreceptor, Env undergoes further conformational changes that expose the fusion machinery located in the gp41 subunit and, particularly, a highly hydrophobic region (hidden in the native protein), the fusion peptide (FP), is inserted into the host cell membrane, triggering the membrane fusion process (Figure 2c). The gp41 regions heptad repeats 1 and 2 (HR1 and HR2) sequences and the Membrane Proximal External Region (MPER) play a pivotal role in this fusion process [19].

CD4 is expressed at high density on the surface of CD4 T cells and at a lower density on other immune cells, such as monocytes, macrophages, and dendritic cells (DC) [22] defining the main targets for potential infection. The expression of coreceptors further delineates cell target tropism. Whereas CXCR4 is widely expressed in several cell types, CCR5 expression is restricted to a subset of memory CD4 T cells and myeloid cells [23,24]. Wider expression of CXCR4 makes X4 tropic viruses more cytopathic and associated with faster disease progression [25]. However, most transmitted viruses show an R5 phenotype and efficiently infect CCR5+ CD4 T cells, while infection of macrophages requires higher gp120/CD4 binding affinity and is restricted to the so-called macrophage-tropic HIV isolates [22].

Once the viral membrane has fused with the target cell, the HIV-1 capsid containing the RT and IN viral enzymes [18] is released into the cytoplasm of the infected cell. Those enzymes are coded by pol and are responsible for the reverse transcription of the ssRNA to double-stranded deoxyribonucleic acid (dsDNA) and the integration of the retrotranscribed DNA into the host cell (Figure 2c).

The understanding of the process of reverse transcription and uncoating of the newly formed proviral DNA has changed over the last years. The original concept of a rapid disintegration of the viral capsid after entering the cytoplasm has been abandoned. Instead, it is now well described that an intact viral capsid traverses the cytoplasm, reaches the nuclear membrane, and enters the nucleus [26] aided by the Cleavage and Polyadenylation Specificity Factor 6 (CPSF6) host factor. The capsid hijacks CPSF6 and exploits it to access transcriptionally active gene-rich regions of the genome [27]. Through this process, the viral capsid serves as a cloak of invisibility, dissimulating the viral genetic material and protecting the process of reverse transcription against innate immune sensors such as cyclic GMP-AMP synthase and ultimately permitting the delivery of the HIV proviral DNA inside the nucleus [28] (Figure 2c).

Since the RT is a low-fidelity enzyme prone to mismatches and does not have proofreading activity, the mutational rate of HIV-1 is notably high (approximately 3 × 10^−5^ mutations/bp/cycle), resulting in the introduction of one mutation throughout the 10 kb every 3 cycles and hundreds of newly mutated viruses each time a cell is infected [29]. This high mutational rate, combined with recombination events, results in a constellation of viral sequences within one individual that comprise a quasispecies [30], and leads to a quick evolution of the virus when it faces selective pressure from treatments or the immune system [31].

Upon reverse transcription, viral DNA integrates into the host cell’s genome. The integration process is led by the viral enzyme integrase but is a quite complex event requiring multiple cellular cofactors to allow for the cleavage of chromosomal DNA, the transfer of the proviral DNA sequence and finally the repair of the DNA nick. Among these factors, lens epithelium-derived growth factor (LEDGF/p75) and DNA repair enzymes are critical [32].

Once the proviral DNA has been integrated into the host cell genome, different potential outcomes are possible in association with the integration site and the activation status of the infected cell: in non-activated cells, HIV-1 provirus can remain silent for years, allowing for the establishment of a latent viral reservoir [33].

The first HIV-1 genes synthesised in the cytoplasm are the regulatory genes *tat* and *rev*. Tat (transactivator of transcription) acts as a transcription factor for the expression of HIV genes by binding to the transactivation response (TAR) sequence of the HIV LTR [34], while Rev facilitates the nuclear export of unspliced and partially spliced viral mRNAs by binding to the tertiary RNA structure rev-responsive element (RRE) [35]. Both events promote the expression of the structural protein p55Gag (Gag), the Pr160GagPol or Gag-Pol polyprotein, and Env. Even though Gag and Pol are coded in different Open Reading Frames (ORFs), the presence of a slippery sequence promotes a ribosomal frameshift that allows for the synthesis of the Gag-Pol polyprotein [36]. Gag and Gag-Pol are synthesised in the cytosol at a 20:1 ratio [37] and associate with the inner leaflet of the plasma membrane after palmitoylation to facilitate oligomerisation and budding [38]. Gag is necessary and sufficient to support the formation of virion particles. In addition, Gag mediates the packaging of the viral RNA genome as well as the viral accessory protein Vpr inside the newly forming virion [39]. In parallel to these events, Env is synthesised inside the endoplasmic reticulum and is transported through the Golgi Apparatus to the plasma membrane, where it is incorporated at a low density on the surface of the virion [40]. Some host cell proteins expressed at the surface of the cell can also be actively or passively incorporated into the viral particle [41]. Finally, the virion buds from the plasma membrane of the host cell, a process that require the endosomal sorting complexes required for transport (ESCRT) machinery [42], whilst the protease (PR) within Pol mediates its auto processing and the proteolysis of the Gag and Gag-Pol polyproteins into the final functional structural elements (the matrix or MA, the capsid or CA and the nucleocapsid or NC) and the viral enzymes reverse transcriptase (RT) and integrase (IN), resulting in the maturation of the virion into a fully infectious HIV-1 particle [43] (Figure 2c).

### 2.3. Cell-to-Cell Transmission

Although the classical view of the HIV life cycle is associated with cell-free virus infection, the spread of HIV In Vivo could, in fact, be mostly mediated by cell-to-cell mediated mechanisms [44]. HIV hijacks the intercellular communication mechanisms of the immune system to efficiently infect CD4 T cells. Two main mechanisms have been described with different outcomes. In the first one, HIV exploit DCs as a vehicle to access areas enriched in CD4+ Th lymphocytes, such as the lymph nodes, through a mechanism defined as “transinfection” [45]. DCs are the first cells to detect HIV-1 presence through pattern recognition receptors (PRR) such as toll-like receptors (TLR) and sialic acid-binding immunoglobulin-type lectins (Siglec) [46]. These professional APCs become activated upon viral recognition and phagocytosis of the pathogen, especially in the mucosal barrier and migrate to the lymph nodes, where they present HIV-1-derived antigens to lymphocytes, activating antigen-specific adaptive immune responses. During this process, captured intact particles can also be transferred from DCs to target cells efficiently infecting and activating them (Figure 2d). Although antigen presentation is not strictly required for transinfection, T-cell activation greatly increases in productive infection and unsurprisingly, HIV-specific activated CD4+ T cells are the main target of HIV-1 infection [47].

A second mechanism involves direct contact between infected and uninfected CD4 T cells. These contacts are not necessarily inducing CD4 T-cell activation, but a massive attack of viruses that trigger different pathways of danger detection by the innate cellular surveillance, resulting in cell death [44]. Indeed, these so-called virological synapses [48] have been associated with apoptotic, autophagic and pyroptotic CD4 T cell death, thus potentially contributing to CD4 T-cell depletion [49,50,51,52] (Figure 2d). Furthermore, cell-to-cell fusion events may also occur, contributing to HIV spread [53] and cytopathogenicity [54]; even though their role in vivo is still unclear [55].

### 2.4. Accessory Genes and Cellular Restriction Factors

Other than *tat* and *rev*, the HIV genome encodes for additional accessory genes (*vif*, *vpr*, *vpu* and *nef*), whose study has revealed multiple functions and has helped to identify a plethora of innate cellular antiviral mechanisms defined collectively as host restriction factors, which are usually induced by Type I IFN responses [56]. Complementarily, the study of SIV and HIV-2 viruses has provided a full understanding of the complex steps involving retroviral replication [57]. Some of which are summarised in this section.

Vif stands for viral infectivity factor and was known to be necessary for HIV infectivity in otherwise refractory cell lines. Its cellular counterpart was identified as the Apolipoprotein B mRNA editing enzyme, catalytic polypeptide-like 3G (APOBEC3G) [58]. APOBEC3G is a cytidine deaminase that introduces mutations in viral RNAs, leading to non-viable viral genomes and protection from subsequent infection. However, Vif interacts with APOBEC3G, promoting its polyubiquitination and proteasomal degradation.

Vpr, which stands for Viral protein R, is the only HIV accessory protein packaged in high quantity inside the virions, since it is active during the early infection steps. Although its precise function remains enigmatic, Vpr has been shown to interact and interfere with several cellular pathways. Notably, Vpr degrades the nuclear protein CCDC137/cPERP-B, allowing for the arrest of the cell cycle of infected cells at the G2/M phase, though, in which the viral transcription seems to be optimal [59]. In addition, other host restriction factors such as RNA-associated early-stage antiviral factor (REAF), TET2 or IRF3 can also be targeted by Vpr [56].

The study of Vpu (Viral Protein U) revealed the existence of an additional host restriction factor, Tetherin or BST-2. This protein binds to the lipidic viral envelope and to the cellular plasma membrane, preventing the release of viral particles and therefore limiting viral spread [60] but conversely, Tetherin cell surface expression is downregulated by Vpu [61]. Additionally, Vpu also contributes to CD4 downregulation, which is necessary to avoid intracellular fusion events in infected cells and to maintain the infectivity of viral particles [62].

Finally, Nef (Negative Regulatory Factor) is a multifunctional protein that associates with the plasma membrane through myristylation and interacts with adaptor proteins AP-1 and AP-2 [63,64]. Nef downregulates CD4 expression to facilitate HIV infectivity [64], and Major histocompatibility class I molecules, to reduce viral peptide presentation to CD8 T cells and protect infected cells from the immune response [64]. Nef has been linked to cholesterol transport to the plasma membrane, a key event, as cholesterol is a necessary lipid in the viral envelope [65]. Finally, Nef regulates the expression of several proteins that inhibit membrane fusion during viral entry, such as Serin-5 and IFN-induced trans membrane proteins (IFITMs) [56,66].

The coordinated action of these accessory proteins enables HIV to efficiently infect human cells, spread among them, and promote human-to-human transmission, thus being determinant for HIV pathogenesis.

## 3. Pathogenesis of HIV Infection

### 3.1. Acute HIV Infection

**Figure 3:**
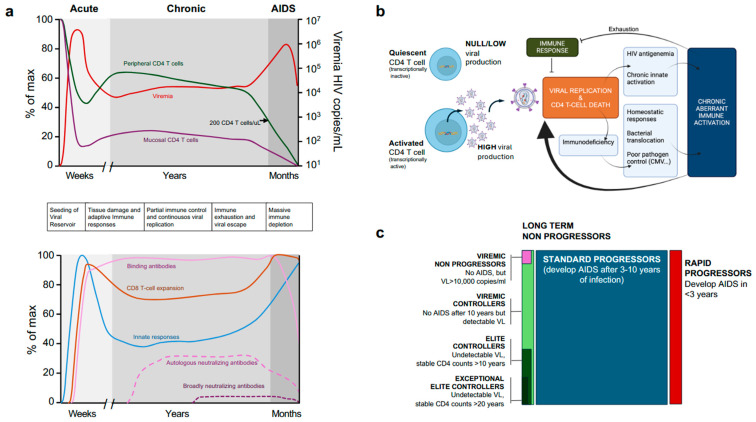
Main events occurring after HIV infection. (**a**) Dynamics of main clinical parameters (plasma viral load/Viremia and peripheral CD4 T cell counts) along with mucosal CD4 T cells (**Top**) and dynamics of innate and adaptive immune responses (**Bottom**). The phases of infection are depicted in different grey tones, and the main events are sequentially described between the two figures. (**b**) Main event controlling virological setpoint in the chronic phase of HIV infection and main causes for viral escape (see text for details). (**c**) Main clinical phenotypes of untreated HIV infection, with description of each one. The rectangular surface approximately indicates the frequency of each phenotype.

HIV is mostly transmitted by sexual intercourse (estimated to be responsible for 80% of infections), by direct blood contact (associated with intravenous drug users) and by vertical transmission from infected mothers to newborns (either intra-utero, at delivery or postpartum by breastfeeding [67]. In most cases, HIV pathophysiological consequences could be unnoticeable until they reach an irreversible point in which the host is in a deep immunosuppression state (AIDS) and opportunistic infections easily arise. HIV-1 infection can be broadly divided into three main stages: (i) acute infection, (ii) chronic infection, and (iii) AIDS (Figure 3a).

During primoinfection, HIV locally spreads at mucosal sites and is efficiently transported to lymph nodes by DCs, leading to the transinfection of tissue CD4+ T cells. From there, the virus can reach secondary lymphoid organs such as the Gut-Associated Lymphoid Tissue [68]. In these CD4+ rich tissues, HIV can multiply while latently infecting and establishing a notorious viral reservoir [69]. After a mean [range] of 13 (6–18) days, viremia reaches a peak of 6.7 (4.5–8.5) log10 copies/mL of blood, which results in a moderate drop in circulating CD4+ T-cell counts [70] (Figure 3a).

This high viral load triggers immune responses to control the viral replication. Initially, there is a striking cascade response of acute phase reactants and inflammatory cytokines that has been referred to as the ‘cytokine storm’ [71,72]. Studies in this area have demonstrated inconsistencies in the type and timing of cytokine observations, including reports of significant upregulation of cytokines which are not observed or contested by others [72,73,74,75,76,77]. The discrepancies may be due in part to differences in the cytokine measurement assays, but likely also reflect the highly dynamic nature of AHI in a short time frame, thus leading to high variability between cross-sectional samplings [78]. The very first measurable innate immune response, detectable just prior to the appearance of plasma HIV-1 RNA, is the acute phase protein serum amyloid A [77]. Thereafter, levels of interleukin (IL)-15, type I interferon (IFN)-α and interferon gamma inducible protein 10 (CXCL10/IP-10) are increased rapidly, followed by an increase in IL-10, IL-18, tumour necrosis factor (TNF) and IFN-γ [71,72,73,74,75,76,77]. The cellular sources of these acute-phase cytokines have not been definitively identified, but most likely include infected CD4 T-cells and innate cells such as plasmacytoid DCs [79]. Some cytokine storm components, including IL-12, IFN-γ, IP-10, IL-7 and IL-15, can predict VL set point, T-cell activation and CD4 T-cell count or the subsequent disease progression, as shown for both HIV infection in humans and SIV infection in NHP [76,77,78,79,80].

The first detectable humoral response after HIV infection is in the form of immune complexes around eight days after plasma virus detection, whereas the first free plasma anti-HIV antibodies appear 13 days after detectable plasma viremia. These antibodies are from the IgM isotype and are directed against the Env subunit gp41, which will be followed by IgG and IgA isotypes [81]. gp120-specific antibodies take an additional 14 days [82]. IgG antibodies against inner viral proteins appear at a median time of 18 days (CA, GAG) and 33 days (MA) following detectable plasma HIV-1 RNA [81], while antibodies against the Integrase protein are the last elicited, appearing at a median time of 53 days after detectable viremia [81]. Antibodies that neutralise the transmitted founder virus and select for viral escape are not detected until at least three months after infection and are mainly directed to Env variable regions [83,84], accounting for the extensive variation in the env gene that is observed in early HIV infection [83,84,85]. However, antibodies that show some degree of neutralisation of heterologous virus eventually arise, years after infection, in only 10–25% of patients [86,87], and highly potent broadly neutralising antibodies active against a large diversity of viral isolates, are only identified in the so-called ‘elite neutralisers’, which represent an estimated 1% of total HIV infected individuals (Figure 3a) [88,89].

HIV specific T-cell responses appear concomitantly to antibodies; both CD4 and CD8 T cells show signs of extensive activation and a progressive loss of resting cells. Expansion of HIV specific T cells reaches a peak three weeks after infection and can be traced by cell-surface expression of CD38 and the human leukocyte antigen-D related (HLA-DR) markers on CD4 and CD8 T cells [90]. Activated T cells show a high level of activation-induced apoptosis 46, a condition reflected by the expression of exhaustion cell markers like Programmed Cell Death protein-1 (PD-1), Tim-3 and Lag-3 proteins, which were shown to be predictive of disease progression [90,91]. Polyfunctional CD8 T cell responses, particularly against Gag, seem to be mainly responsible for immune control of HIV replication [92].

Eventually, initial innate responses and subsequent cytotoxic CD8+ T lymphocytes responses and antibodies can partially control viral replication, which reaches after 31 days (18–42) a heterogeneous stable set point of viraemia 4.4 (2,5-6.0) log10 copies/mL [93]. The viral setpoint is defined by the individual balance between viral replication and immune responses [71].

Other than viral replication and immune responses, two main events are associated with primoinfection. Firstly, upon infection of CD4+ T cells, HIV proviral genomes integrate into host cell DNA, remaining silent and leading to the creation of a latent reservoir, which is seeded early after primoinfection. Furthermore, the HIV-1 reservoir can grow through the clonal expansion of a CD4+ T cell that harbours the HIV-1 proviral DNA integrated in its genome. Activation and duplication of these cells may lead to an increase in the viral reservoir without the need for active replication [94]. A second relevant event is the selective and profound depletion of CCR5+ CD4 T cells in the GALT [95,96], which will remain severely and irreversibly damaged during the course of infection [97] compromising the impermeability of the gastric epithelium and favouring bacterial translocation to the bloodstream. Consequently, levels of plasma LPS are elevated in HIV infected individuals, resulting in chronic activation of TLR4 in innate immune cells [98].

Importantly, during this acute phase, mild flu-like or unnoticed symptomatology is reported; consequently, in most cases, HIV tests are not performed, and most primary infections remain undiagnosed, fuelling transmission.

### 3.2. Chronic HIV Infection and AIDS

Reaching a stable viral set point will mark the end of the acute phase and the beginning of the chronic phase. Again, this is a clinically silent phase, in which the infected individual maintains a suboptimal immune function that still allows pathogen control. However, this is a highly dynamic phase in which the virus continuously replicates and destroys CD4 T cells, while the immune system tries to compensate for cell destruction and to limit viral replication by maintaining a high level of CD8 T cell activation and inflammation (Figure 3a) that can lead to exhaustion. HIV-1 can eventually escape immune control, and the CD4+ T cell count will slowly but steadily decrease below 200 CD4 T cells/µL (the threshold definitive of AIDS), establishing the final AIDS phase, which can take from months to several years to develop. In the AIDS phase, opportunistic pathogens can infect the host, leading to death [99].

A summary of the HIV infection chronic phase is shown in Figure 3b. Each infected individual reaches a set point at which viral replication and immune responses are balanced. However, viral replication induces antigenemia and adaptive immune system activation (both cellular and humoral). These signals are complemented by innate sensing of HIV replication and chronic Type I interferon responses [100]. In parallel, partial CD4 T cell immunodeficiency is compensated for by increased CD4 T cell proliferation [101], and impairs GALT barrier function, leading to increased bacterial translocation [97], which in turn activates innate sensing. Furthermore, a potential poor control of other chronic infections (such as CMV or EBV) can also contribute to immune activation [102]. This aberrant and chronic immune activation has two main detrimental effects: fuelling viral replication (which is dependent on T-cell activation) and exhausting immune responses, both contributing to viral escape and AIDS [103] (Figure 3b).

During the chronic phase of infection, HIV-1 can exploit their mechanisms of spread and the lack of full control by the immune system to access far-off tissues like the central nervous system (CNS). HIV crosses the blood–brain Barrier (BBB) with the aid of monocytes [104] and remains in the CNS beyond the influence of the immune system, effectively establishing a reservoir within this “sanctuary”. Neurological complications of HIV-1 infection have been widely reported and associated with viral proteins Tat and Env, leading in some cases to HIV-associated dementia (HAD) [105].

### 3.3. Clinical Consequences and Progression Phenotypes

Untreated HIV infected individuals progress to AIDS in a temporal range of 3–10 years after primo-infection. It is accepted that lower CD4+ T-cell counts and high viral setpoints result in faster disease progression, while higher CD4+ T-cell counts and lower viral setpoints generally lead to a slower progression of the disease [70]. However, less than 10% of untreated patients fall into different groups: rapid progressors or long-term non-progressors, which can be divided into viremic non-progressors showing high viremia in the absence of disease progression, a rare phenotype particularly found in the paediatric population [106]; viraemic controllers, when the viral setpoint is established below 2000 copies/mL; or elite controllers, that show undetectable viral load and stable CD4 T cell counts for more than 10 or 20 years (exceptional elite controllers) (Figure 3c) [107].

The study of these extreme phenotypes has provided information on the factors controlling HIV pathogenesis. Rapid progression is associated with X4 tropism and low immune responses. Particularly, non-seroconversion after primoinfection is associated with ultra-rapid progression and death [108,109]. In contrast, some group B HLA alleles are the most notorious predictors of long-term non-progressor and elite controller profiles, namely HLA B*27, B*57, and B*58 [110,111]. Furthermore, HLA-II molecules (such as DRB1*12:01) have been linked with lower viral loads and controller profiles [112]; and effective CD4+ Th and CD8+ Tc responses against Gag have been clearly associated with a slower disease progression [113]. Another feature associated with natural protection is a 32 bp deletion in the CCR5 coreceptor (CCR5Δ32), since coreceptor usage is essential for HIV-1 to infect a CD4+ T cell [114]. Homozygous CCR5Δ32 individuals are naturally resistant to R5 HIV-1 infection and have been used as donors of haematopoietic stem cells for PLWH who required a bone marrow transplant due to leukaemia, resulting in the three HIV-1 sterilising cures described to date: the Berlin Patient, the London Patient, and the Dusseldorf Patient [115].

Several viral characteristics have been associated with long-term non-progression, including deletions in the *nef* gene that impair viral replication [116], or the presence of poorly functional env genes with poor fusogenic activity and poor affinity for CD4 [117,118,119]. Therefore, it seems that a combination of poor viral fitness and potent immune responses could lead to an Elite Control setting, the natural “functional cure” of HIV infection. The study of this population can help inform us about the features associated with protective immune responses, which could be useful for the development of better vaccines and immunotherapies aimed at HIV eradication [120].

## 4. Diagnostic and Treatment

### 4.1. Diagnosis of HIV Infection

Due to the silent symptomatology of HIV infection, HIV diagnosis tools are essential to identify HIV infection and provide appropriate linkage to care for infected individuals, stopping both immune damage at the individual level and new transmissions at the community level. The most common tests are based on serology or on the sensitive detection of viral RNA by Nucleic Acid Tests (NAT). According to the sensitivity of the different tests, primo-infection events can be divided into six stages (Fiebig I–VI) [121], corresponding to the sequential appearance of detectable levels of viremia and different types of antigens and antibodies in plasma samples.

Serological assays have evolved with the pandemic, and currently available tests include highly sensitive fifth-generation tests for the detection of p24/CA antigen and IgM/IgG antibodies by sandwich ELISA technology [67]. According to the WHO, diagnosis strategies require a confirmatory test after the first screen with a highly sensitive method. Confirmatory tests should be made with a different technique, which usually involves the analysis of the reactivity of antibodies against the major structural viral proteins (env, gag and pol subunits) [122].

Other than laboratory tests, the need for extended HIV testing in high HIV burden areas or communities has been fulfilled by the development of point-of-care (PoC) tests. Although less sensitive than laboratory assays, they are simpler, cheaper and contribute to improved testing coverage [123]. Confirmatory tests are also needed after a positive PoC result.

In addition to diagnosis, the clinical follow-up of HIV infected individuals is based on the monitoring of both plasma Viral Load and the number of circulating CD4 and CD8 T cells. Currently, RT-PCR-based methods offer a sensitivity of 20 RNA copies/mL of plasma. Although ultrasensitive methods going down to 1 RNA copy/mL have been developed, they are not used in clinical routine. Quantification of circulating T cells is performed by standard flow cytometry using anti-CD3, CD4 and CD8 antibodies, and provides also information on the CD4/CD8 T cell ratio that represents a clinically relevant parameter to monitor disease progression [124].

### 4.2. The Era of Antiretroviral Drugs

In the past 30 years, major advances in HIV-1 care came by the hand of antiretroviral drugs, leading to the current situation in which people who have access to antiretroviral therapy (ART) and adhere to the treatment do not develop severe complications associated with HIV-1 chronic infection, unless escape mutations arise.

The first antiretroviral drug against HIV arrived in 1987, when zidovudine (AZT) was approved by the FDA to treat AIDS. AZT was a big step forward in HIV treatment, since it targeted the HIV reverse transcriptase and reduced AIDS-related mortality of people with HIV [125]. In the following years, similar treatments such as didanosine, zalcitabine and stavudine were approved by the FDA, but escape mutants rapidly emerged [126], and in 1995–1996, drugs targeting other viral pathways were developed [127], leading to the possibility of combining antiretrovirals and starting the era of highly active ART (HAART) [128].

All the current therapies approved for HIV-1 treatment are mainly small molecules that target different elements of the virus [129]. These drugs are divided into several classes according to their HIV-1 target: nucleoside reverse transcriptase inhibitors (NRTI), non-nucleoside reverse transcriptase inhibitors (NNRTI), protease inhibitors (PI), integrase strand transfer inhibitors (INSTI), and the recently approved Capsid inhibitors [130]. Other targets, such as fusion inhibitors and CCR5 antagonists, have also been developed [131]. The standard of care for ART states that all HIV-1-infected individuals must be treated with a combination of three drugs using at least two different drug classes [132].

The greatest success of ART is that it achieves undetectable viral loads in the blood of PLWH, minimising the risk for the emergence of resistant mutated viruses and keeping the disease progression at bay, unless the therapy is discontinued. Still, one major ART challenge is poor biodistribution, especially in HIV-1 sanctuaries (e.g., CNS), in which the virus can maintain a low level of transcriptional activity that leads to neuroinflammation, and could eventually lead to the emergence of ART-resistant mutations [133]. Hence, early treatment with ART also results in virological and immunological benefits, since the reservoir is smaller and CD4+ Th cells are better preserved [134].

Not only have antiretroviral drugs achieved a strict control of the viraemia in PLWH, but they have also proven effective at preventing infection in populations at risk or already exposed to the virus by pre- or postexposure prophylaxis (PrEP and PEP) interventions, respectively [135]. Truvada, a combination of emtricitabine and tenofovir (both reverse transcription inhibitors), has been approved for its use as a PrEP agent. PrEP is currently living a revolution with the recent development of long-acting integrase inhibitors (Cabotegravir-LA) and long-acting capsid inhibitors (such as Lenacapavir), which can contribute to optimising these interventions [136,137].

Despite all these major successes in the control and prevention of HIV-1 infection by antiretroviral drugs, no pharmaceutical intervention has yet succeeded in achieving a functional cure for PLWH. That is why new therapies with anti-reservoir activity, such as CAR T-cells or engineered antibodies, are being extensively developed and evaluated [138].

## 5. The Impact of ART on HIV Infection

**Figure 4:**
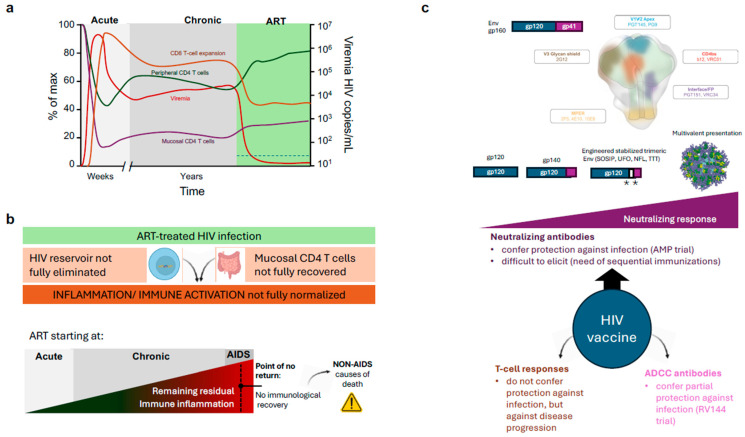
Therapies and Vaccines against HIV infection. (**a**) Impact of standard Antiretroviral therapy (ART) on plasma viral load (viremia), Peripheral and mucosal CD4 T cell counts and CD8 T cell activation/Expansion. The ART period is highlighted in green, and the dotted line on the bottom left indicates the limit of detection of current viremia tests. (**b**) Main limitations of ART. Lack of impact on the reservoir and GALT CD4 T-cell recovery maintains an abnormal level of inflammation/immune activation in treated HIV infected individuals. This level is increased when treatment is delayed and may achieve a “point of no return”, leading to a blunted CD4 T cell recovery and increased risk of death. (**c**) Main HIV vaccine strategies are focused on the development of improved immunogens and delivery to develop broadly neutralising antibodies against the indicated Env regions. Immunogens are schematically represented; the white area between gp120 and gp41 represents linkers, and asterisks represent engineered mutations (**Top**). Other strategies based on T cell responses and ADCC-promoting antibodies are shown (**Bottom**).

ART induces a decline in viral replication and an increase in CD4 T-cell counts, and consequently a reduction in T-cell activation and cell death. Viral load in plasma becomes rapidly undetectable using standard techniques (<20 copies/mL) (Figure 4a). In parallel, the size of the HIV reservoir is reduced but is not eliminated (Figure 4b). Reduction follows a biphasic decay kinetics to reach a plateau after 2 months on ART [139]. The plateau has a slightly negative slope, probably associated with spontaneous reservoir reactivation and elimination, but it has been calculated that a reduction in the HIV reservoir by ART would require more than 60 years [140]. In addition, clonal expansion of latently infected cells has been described in some individuals, leading to increased reservoir size over time [141].

On the immunological side, an adequate CD4 T-cell recovery on HAART is defined as a biphasic response with an initial fast increase in CD4 T-cells, followed by a slower repopulation phase. The accelerated gain of CD4 T-cells in the first months of treatment (20–30 cells/μL/month) is primarily due to a reduction in apoptosis and a redistribution of previously trapped memory T-cells from lymphoid tissue, where the replication was taking place, to the bloodstream [142]. In contrast, the sustained recovery is generated mainly by naïve T-cells. Various mechanisms may account for this rise: first, *de novo* production of T-cells by the thymus; second, the homeostatic proliferation of the residual CD4 T-cells; and third, the extension of CD4 T-cell half-life, a mechanism responsible for sustaining the number of naive CD4 T-cells in older individuals. However, as mentioned above, the recovery of circulating CD4 T cells does not parallel the situation of tissues (such as GALT), in which the recovery of CD4 T cells is minimal and partial immunodeficiency is permanent (Figure 4b).

Both the lack of mucosal CD4 T cells and the persistence of latently infected cells that may produce viral particles or viral antigens, contribute to the lack of normalisation of the immune activation at the innate level (Type I IFN responses) and at the adaptive level (CD8 T-cell activation) in treated HIV infected individuals [143]. A wide range of inflammatory markers have been described to be altered and associated with comorbidities, such as cardiovascular disease risk or inflammaging [144]. As a rule, residual inflammation in treated HIV increases when ART is started in late disease phases. Importantly, it is well described that 15–40% of individuals starting ART below 200 CD4 T cells/µL will not recover the number of circulating CD4 T cells despite completely suppressing viral replication (Immunodiscordant or immunological non-responder individuals) [145]. Although the reasons for this lack of peripheral immune recovery are unclear [146,147], this observation suggests that there is a “point of no return” in the immunological damage induced by HIV. This is clinically relevant, since immunodiscordant individuals are at higher risk of AIDS and non-AIDS-related death [148] (Figure 4b).

## 6. Vaccines, the Unmet Need

### 6.1. Identifying Protective Immune Responses Against HIV

Neutralising antibodies (NAbs) against HIV-1 arising after HIV primoinfection show autologous neutralisation against the Env expressed by the transmitter/founder virus and rarely exhibit heterologous neutralisation against other clades; however, the selective immune pressure they exert on the virus will end up prompting the expansion of mutated escape variants [149]. The immune system adapts in parallel to HIV evolution, generating new antibodies and T-cell responses, or improving by somatic hypermutation (SHM) the preexisting antibody responses. Remarkably, years after infection, around 1% of the PLWH develop exceptional broadly neutralising antibodies (bNAbs) that neutralise a wide range of HIV-1 variants [89].

Structurally speaking, bNAbs display some general features independently of the Env epitope they target [150]. First, bNAbs target conserved regions of Env, namely the CD4bs, the V1V2 apex, the MPER, the Fusion Peptide (FP) or the gp120/gp41 interface (Figure 4c). On the molecular level, bNAbs display a considerably high SHM rate (40–100 mutations compared to the 10–20 mutations of standard antibodies), proving how bNAbs undergo several cycles of SHM and antigen selection at the Germinal Centre (GC) throughout many years of being exposed to an evolving antigen [151]. Furthermore, these mutations are more frequently located in the Complementary Determining Regions (CDRs) but also at the framework areas of the variable domain, which are generally stringent to mutations [152]. Another exceptional feature of bNAbs is the unusually long CDRH3 domains. CDRH3 loops in regular antibodies are 8–16 aa-long, while bNAbs on average have 21 aa-long CDRH3 fingers [153].

Although the role of bNAbs in the control of viral replication and disease progression in HIV-1-infected individuals is controversial [154], they have recently proven to be a potentially successful strategy to mediate a protective effect or to be used as a treatment by passive immunisation in Antibody-Mediated Protection (AMP) strategies. Initial studies in animal models demonstrated the potential of this approach both in mice [155] and macaques [156], especially when used in combination with treatments of two or more bNAbs with different specificities [157]. AMP has also been tested in human clinical trials. VRC01, an anti-CD4bs bNAb, was tested for its capacity to mediate protection in populations at risk of HIV acquisition in two different phase IIb clinical trials: HVTN703/HPTN081 and HVTN704/HPTN085. Preliminary results of both trials showed that VRC01 was safe and tolerable but did not show significant protective effects [158]. However, it is relevant to highlight that VRC01-treated people did achieve significantly more infections with VRC01 neutralisation-resistant viruses than placebo groups, suggesting that protection could be mediated by passive immunisation using a combination of broader and more potent bNAbs [158].

The antiviral effects of bNAbs are clear, and their potential is not only limited to AMP and treatment, but they could also play a key role in targeting HIV-1 reservoirs in the pursuit of a functional cure [159]. Results derived from bNAbs’ research are also informative for the development of better vaccine strategies.

Abs can mediate protection beyond the direct neutralisation of HIV-1 by inducing antibody-dependent effector functions such as antibody-dependent cellular cytotoxicity (ADCC) (Figure 4c). The best example is the RV144 trial —also known as “Thai Trial”—(clinicaltrials.gov: NCT00223080). In this study, a canarypox vector (vCP1521), named ALVAC-HIV, coding for a subtype B gag-pol and a membrane-bound CRF01_AE gp120 linked to a subtype B gp41, was administered four times, and alum-adjuvanted AIDSVAX B/E was administered twice, together with the third and fourth ALVAC-HIV doses [160]. Despite a wave of criticism for choosing vaccine components that did not demonstrate In Vivo efficacy in previous human trials separately [161], RV144 demonstrated an overall protection of 31% positively associated with antibodies targeting the V1V2 apex [162]. These anti-V1V2 antibodies were not able to neutralise the virus but mediated ADCC [163]. Unfortunately, the subsequent similar trial, HVTN702, was halted due to futility detected in an interim analysis in early 2020.

Considering cellular responses, Env has been widely reported as a poor stimulator of protective CD4+ Th and CD8+ Tc lymphocytes, while Gag-Pol polyprotein and accessory proteins, like Nef, are better targeted by the cellular arm of the immune response. However, T-cell responses are not associated with protection against infection, but against disease progression (Figure 4c) [164].

### 6.2. HIV-1 Candidate Immunogens for Vaccine Development

HIV-1 immunogens developed for vaccination include those designed for the induction of potent cellular responses and those that aim at eliciting protective humoral responses. Some examples of the former immunogens are mosaic proteins —bioinformatically designed Gag proteins made up of different fragments from multiple subtypes that cover most HIV-1 group M clades [165,166]—, HIV.consv—encoding only for the most conserved elements of Gag—, or HTI—encoding for immunogens that express conserved peptides targeted by effective cellular responses [164].

In comparison, Env is the main target of neutralising antibodies. Immunisation with unmodified Env proteins (soluble gp120 or g140 forms or vectored vaccines, AIDSVAX or ALVAC, respectively) are capable of eliciting robust antibody responses [167]; however, it has proven to be insufficient to generate a protective bNAb response [168,169,170]. More sophisticated immunogens have been engineered to present key epitopes targeted by bNAbs (Figure 4c). These immunogens present trimeric Env in a stable native conformation, a strategy that has thrust the research on bNAb-like response induction [171]. Env trimers are mainly categorised into three groups: NFL trimers—native flexible linked—, UFO trimers—uncleaved prefusion optimised— and SOSIP—soluble-stabilised gp140 with I559P [172]. SOSIP trimers contain multiple stabilising mutations: disulphide bridges between gp120 and gp41, a stabilising I559P point mutation, and a truncation at amino acid 664, which confers them similar antigenicity as membrane-bound viral trimeric Env [173]. SOSIP trimers have been generated using different sequences of HIV-1 Env, including primary isolates like JRFL [173], then adapting the platform to BG505 a subtype A transmitter/founder strain [174], and more recently, based on ConS and ConM that contain consensus HIV-1 sequences [175], or GT1.1 that was designed to specifically elicit VRC01-like responses [176]. Some of these immunogens have successfully completed Phase I safety trials (NCT03699241, NCT04224701, NCT04177355, NCT03961438, NCT03816137).

Another strategy to increase the immunogenicity of subunit proteins is their administration, formulated or conjugated with a multivalent platform or with improved delivery systems [177]. Multivalent platforms come in a wide range of sizes, structures and compositions [178,179], but share an increased immunogenicity and a better kinetics of immune responses. Such is the case of the lumazine-based eOD-GT8 prototype, a vaccine formed by 60-mers of an Env engineered Outer Domain (eOD) immunogen that was designed to trigger VRC01-like germline ancestors, an anti-CD4bs bNAb precursor, and which has been tested in phase I clinical trials, achieving the expected outcomes (clinicaltrials.gov: NCT03547245). Other protein-based nanocarriers are ferritin nanoparticles, which can accommodate SOSIP trimers and demonstrate superior neutralising responses in rabbits when compared to the soluble protein [179].

### 6.3. Immunisation Strategies

A successful HIV-1 vaccine will not only come from the identification of immunogens and platforms that elicit potent responses, but also from the characterisation of which platforms, immunogens, and adjuvants synergise better to induce superior neutralising and effector-mediating antibodies together with effective cellular responses [180]. The RV144 trial is a clear example of that, since AIDSVAX, a component that did not elicit protective responses on its own, managed to induce moderate protection in combination with ALVAC-HIV [181].

The current paradigm in HIV vaccine research is the use of sequential immunisation strategies to elicit bNAbs. Due to the scarcity of bNAb precursors and the fact that current immunogens are not able to mobilise them, this paradigm suggests that three sequential steps may be essential for bNAb induction: (i) germline targeting with a priming immunogen, (ii) shepherding with a different Env immunogen that helps antibodies undergo affinity maturation, and (iii) polishing with a native Env molecule.

This strategy is used by the eOD-GT8 immunogen, which is designed to generate CD4bs bNAbs [182], in conjunction with stable trimeric proteins displaying Env’s native conformation. Other epitope-based vaccines try to elicit humoral responses against vulnerable regions like the FP or the MPER [183], while removing more immunogenic but irrelevant domains that can divert the elicitation of protective responses against vulnerable domains.

Overall, the analysis of preclinical and clinical data on HIV-1 vaccine efficacy indicates that the final goal of eradicating HIV-1 through the means of prevention will only be achieved with an extremely rational, biologically informed, and highly collaborative vaccine.

## 7. Geographical and Gender Bias

The prevalence and incidence of HIV infection are highly heterogeneous worldwide, as it is the access to care, including HIV testing and treatment [184]. Sub-Saharan Africa remains most severely affected, with nearly 1 in every 25 adults (4.4%) living with HIV and accounting for about 70% of the people living with HIV and almost 90% of HIV-infected children worldwide. In areas most affected, AIDS has raised death rates and lowered life expectancy among adults between the ages of 20 and 49 by about twenty years [185]. Illnesses related to HIV-induced AIDS remain one of the leading causes of death globally and are projected to continue as a significant global cause of premature mortality in the coming decades. Although important progress has been achieved in preventing new HIV infections and in lowering the annual number of AIDS related deaths, the number of people living with HIV continues to increase, especially in some areas of the world and in vulnerable populations [184].

Worldwide, women represent half of all adults living with HIV, and HIV is the leading cause of death among women of reproductive age [184]. Gender inequalities, differential access to services, and sexual violence increase women’s vulnerability to HIV, and, in heterosexual relationships, women are biologically more susceptible to HIV [186]. Young people (ages 15–24) account for about a third of new HIV infections globally [184], significantly affecting those in their most productive years. Most infections are transmitted heterosexually, and AIDS is thus primarily a sexually transmitted disease. However, men who have sex with men, injecting drug users, sex workers, transgender people, and prisoners are disproportionately affected by HIV in most countries [184].

## 8. Concluding Remarks

HIV infection continues to be a major worldwide health issue 40 years after its discovery. Although the vast research in the HIV/AIDS field has uncovered unexpected mechanisms of host–pathogen interaction that greatly increased our knowledge on virology and immunology, this knowledge did not crystallise in an effective vaccine. In contrast, excellent antiretroviral drugs have been developed but are properly implemented exclusively in high-income countries. Efforts should be made to increase HIV testing and access to treatment in developing countries to reduce transmission events until vaccine and cure research can be mature enough to be implemented.

## Data Availability

Data sharing is not applicable as no new data were generated.

## References

[1] Centers for Disease Control (CDC) (1981). Pneumocystis Pneumonia—Los Angeles. Morb. Mortal. Wkly. Rep..

[2] Barré-Sinoussi F., Chermann J.C., Rey F., Nugeyre M.T., Chamaret S., Gruest J., Dauguet C., Axler-Blin C., Vézinet-Brun F., Rouzioux C. (1983). Isolation of a T-Lymphotropic Retrovirus from a Patient at Risk for Acquired Immune Deficiency Syndrome (AIDS). Science.

[3] Barré-Sinoussi F. (2009). HIV: A Discovery Opening the Road to Novel Scientific Knowledge and Global Health Improvement (Nobel Lecture). Angew. Chem. Int. Ed..

[4] UNAIDS (2025). Fact Sheet—Latest Global and Regional Statistics on the Status of the AIDS Epidemic.

[5] Kalish M.L., Wolfe N.D., Ndongmo C.B., McNicholl J., Robbins K.E., Aidoo M., Fonjungo P.N., Alemnji G., Zeh C., Djoko C.F. (2005). Central African Hunters Exposed to Simian Immunodeficiency Virus. Emerg. Infect. Dis..

[6] Sauter D., Kirchhoff F. (2019). Key Viral Adaptations Preceding the AIDS Pandemic. Cell Host Microbe.

[7] Van Heuverswyn F., Li Y., Bailes E., Neel C., Lafay B., Keele B.F., Shaw K.S., Takehisa J., Kraus M.H., Loul S. (2007). Genetic Diversity and Phylogeographic Clustering of SIVcpzPtt in Wild Chimpanzees in Cameroon. Virology.

[8] Tarres-Freixas F. (2021). HIV-1 Virus-Like Particles Engineered to Display a High Antigen Density. Ph.D. Thesis.

[9] Keele B.F., Van Heuverswyn F., Li Y., Bailes E., Takehisa J., Santiago M.L., Bibollet-Ruche F., Chen Y., Wain L.V., Liegeois F. (2006). Chimpanzee Reservoirs of Pandemic and Nonpandemic HIV-1. Science.

[10] D’arc M., Ayouba A., Esteban A., Learn G.H., Boué V., Liegeois F., Etienne L., Tagg N., Leendertz F.H., Boesch C. (2015). Origin of the HIV-1 Group O Epidemic in Western Lowland Gorillas. Proc. Natl. Acad. Sci. USA.

[11] Joos B., Fischer M., Schweizer A., Kuster H., Böni J., Wong J.K., Weber R., Trkola A., Günthard H.F. (2007). Positive in Vivo Selection of the HIV-1 Envelope Protein Gp120 Occurs at Surface-Exposed Regions. J. Infect. Dis..

[12] Sharp P.M., Hahn B.H. (2008). AIDS: Prehistory of HIV-1. Nature.

[13] Worobey M., Gemmel M., Teuwen D.E., Haselkorn T., Kunstman K., Bunce M., Muyembe J.-J., Kabongo J.-M.M., Kalengayi R.M., Van Marck E. (2008). Direct Evidence of Extensive Diversity of HIV-1 in Kinshasa by 1960. Nature.

[14] Faria N.R., Rambaut A., Suchard M.A., Baele G., Bedford T., Ward M.J., Tatem A.J., Sousa J.D., Arinaminpathy N., Pépin J. (2014). HIV Epidemiology. The Early Spread and Epidemic Ignition of HIV-1 in Human Populations. Science.

[15] Korber B., Muldoon M., Theiler J., Gao F., Gupta R., Lapedes A., Hahn B.H., Wolinsky S., Bhattacharya T. (2000). Timing the Ancestor of the HIV-1 Pandemic Strains. Science.

[16] Hemelaar J. (2012). The Origin and Diversity of the HIV-1 Pandemic. Trends Mol. Med..

[17] Tebit D.M., Arts E.J. (2011). Tracking a Century of Global Expansion and Evolution of HIV to Drive Understanding and to Combat Disease. Lancet Infect. Dis..

[18] Moss J.A. (2013). HIV/AIDS Review. Radiol. Technol..

[19] Chen B. (2019). Molecular Mechanism of HIV-1 Entry. Trends Microbiol..

[20] Huang C., Tang M., Zhang M.-Y., Majeed S., Montabana E., Stanfield R.L., Dimitrov D.S., Korber B., Sodroski J., Wilson I.A. (2005). Structure of a V3-Containing HIV-1 Gp120 Core. Science.

[21] Deng H., Liu R., Ellmeier W., Choe S., Unutmaz D., Burkhart M., Di Marzio P., Marmon S., Sutton R.E., Hill C.M. (1996). Identification of a Major Co-Receptor for Primary Isolates of HIV-1. Nature.

[22] Joseph S.B., Swanstrom R. (2018). The Evolution of HIV-1 Entry Phenotypes as a Guide to Changing Target Cells. J. Leukoc. Biol..

[23] Rubbert A., Combadiere C., Ostrowski M., Arthos J., Dybul M., Machado E., Cohn M.A., Hoxie J.A., Murphy P.M., Fauci A.S. (1998). Dendritic Cells Express Multiple Chemokine Receptors Used as Coreceptors for HIV Entry. J. Immunol..

[24] Ostrowski M.A., Justement S.J., Catanzaro A., Hallahan C.A., Ehler L.A., Mizell S.B., Kumar P.N., Mican J.A., Chun T.W., Fauci A.S. (1998). Expression of Chemokine Receptors CXCR4 and CCR5 in HIV-1-Infected and Uninfected Individuals. J. Immunol..

[25] Gorry P.R., Sterjovski J., Churchill M., Witlox K., Gray L., Cunningham A., Wesselingh S. (2004). The Role of Viral Coreceptors and Enhanced Macrophage Tropism in Human Immunodeficiency Virus Type 1 Disease Progression. Sex. Health.

[26] Kreysing J.P., Heidari M., Zila V., Cruz-León S., Obarska-Kosinska A., Laketa V., Rohleder L., Welsch S., Köfinger J., Turoňová B. (2025). Passage of the HIV Capsid Cracks the Nuclear Pore. Cell.

[27] Rasheedi S., Shun M.-C., Serrao E., Sowd G.A., Qian J., Hao C., Dasgupta T., Engelman A.N., Skowronski J. (2016). The Cleavage and Polyadenylation Specificity Factor 6 (CPSF6) Subunit of the Capsid-Recruited Pre-Messenger RNA Cleavage Factor I (CFIm) Complex Mediates HIV-1 Integration into Genes. J. Biol. Chem..

[28] Müller T.G., Zila V., Müller B., Kräusslich H.-G. (2022). Nuclear Capsid Uncoating and Reverse Transcription of HIV-1. Annu. Rev. Virol..

[29] Abram M.E., Ferris A.L., Shao W., Alvord W.G., Hughes S.H. (2010). Nature, Position, and Frequency of Mutations Made in a Single Cycle of HIV-1 Replication. J. Virol..

[30] Domingo E., Perales C. (2019). Viral Quasispecies. PLoS Genet..

[31] Korber B., Gaschen B., Yusim K., Thakallapally R., Kesmir C., Detours V. (2001). Evolutionary and Immunological Implications of Contemporary HIV-1 Variation. Br. Med. Bull..

[32] Debyser Z., Christ F., De Rijck J., Gijsbers R. (2015). Host Factors for Retroviral Integration Site Selection. Trends Biochem. Sci..

[33] Castro-Gonzalez S., Colomer-Lluch M., Serra-Moreno R. (2018). Barriers for HIV Cure: The Latent Reservoir. AIDS Res. Hum. Retroviruses.

[34] Gotora P.T., van der Sluis R., Williams M.E. (2023). HIV-1 Tat Amino Acid Residues That Influence Tat-TAR Binding Affinity: A Scoping Review. BMC Infect. Dis..

[35] Sherpa C., Grice S.F.J.L. (2020). Structural Fluidity of the Human Immunodeficiency Virus Rev Response Element. Viruses.

[36] Biswas P., Jiang X., Pacchia A.L., Dougherty J.P., Peltz S.W. (2004). The Human Immunodeficiency Virus Type 1 Ribosomal Frameshifting Site Is an Invariant Sequence Determinant and an Important Target for Antiviral Therapy. J. Virol..

[37] Shehu-Xhilaga M., Crowe S.M., Mak J. (2001). Maintenance of the Gag/Gag-Pol Ratio Is Important for Human Immunodeficiency Virus Type 1 RNA Dimerization and Viral Infectivity. J. Virol..

[38] Sundquist W.I., Kräusslich H.-G. (2012). HIV-1 Assembly, Budding, and Maturation. Cold Spring Harb. Perspect. Med..

[39] Jouvenet N., Simon S.M., Bieniasz P.D. (2009). Imaging the Interaction of HIV-1 Genomes and Gag during Assembly of Individual Viral Particles. Proc. Natl. Acad. Sci. USA.

[40] Klein J.S., Bjorkman P.J. (2010). Few and Far between: How HIV May Be Evading Antibody Avidity. PLoS Pathog..

[41] Izquierdo-Useros N., Puertas M.C., Borràs F.E., Blanco J., Martinez-Picado J. (2011). Exosomes and Retroviruses: The Chicken or the Egg?. Cell Microbiol..

[42] Demirov D.G., Freed E.O. (2004). Retrovirus Budding. Virus Res..

[43] Freed E.O. (2015). HIV-1 Assembly, Release and Maturation. Nat. Rev. Microbiol..

[44] Agosto L.M., Uchil P.D., Mothes W. (2015). HIV Cell-to-Cell Transmission: Effects on Pathogenesis and Antiretroviral Therapy. Trends Microbiol..

[45] Perez-Zsolt D., Cantero-Pérez J., Erkizia I., Benet S., Pino M., Serra-Peinado C., Hernández-Gallego A., Castellví J., Tapia G., Arnau-Saz V. (2019). Dendritic Cells from the Cervical Mucosa Capture and Transfer HIV-1 via Siglec-1. Front. Immunol..

[46] Perez-Zsolt D., Martinez-Picado J., Izquierdo-Useros N. (2019). When Dendritic Cells Go Viral: The Role of Siglec-1 in Host Defense and Dissemination of Enveloped Viruses. Viruses.

[47] Douek D.C., Brenchley J.M., Betts M.R., Ambrozak D.R., Hill B.J., Okamoto Y., Casazza J.P., Kuruppu J., Kunstman K., Wolinsky S. (2002). HIV Preferentially Infects HIV-Specific CD4+ T Cells. Nature.

[48] Piguet V., Sattentau Q. (2004). Dangerous Liaisons at the Virological Synapse. J. Clin. Investig..

[49] Blanco J., Barretina J., Ferri K.F., Jacotot E., Gutiérrez A., Armand-Ugón M., Cabrera C., Kroemer G., Clotet B., Esté J.A. (2003). Cell-Surface-Expressed HIV-1 Envelope Induces the Death of CD4 T Cells during GP41-Mediated Hemifusion-like Events. Virology.

[50] Blanco J., Barretina J., Clotet B., Esté J.A. (2004). R5 HIV Gp120-Mediated Cellular Contacts Induce the Death of Single CCR5-Expressing CD4 T Cells by a Gp41-Dependent Mechanism. J. Leukoc. Biol..

[51] Denizot M., Varbanov M., Espert L., Robert-Hebmann V., Sagnier S., Garcia E., Curriu M., Mamoun R., Blanco J., Biard-Piechaczyk M. (2008). HIV-1 Gp41 Fusogenic Function Triggers Autophagy in Uninfected Cells. Autophagy.

[52] Doitsh G., Galloway N.L.K., Geng X., Yang Z., Monroe K.M., Zepeda O., Hunt P.W., Hatano H., Sowinski S., Muñoz-Arias I. (2014). Cell Death by Pyroptosis Drives CD4 T-Cell Depletion in HIV-1 Infection. Nature.

[53] Bracq L., Xie M., Benichou S., Bouchet J. (2018). Mechanisms for Cell-to-Cell Transmission of HIV-1. Front. Immunol..

[54] Ferri K.F., Jacotot E., Blanco J., Esté J.A., Zamzami N., Susin S.A., Xie Z., Brothers G., Reed J.C., Penninger J.M. (2000). Apoptosis Control in Syncytia Induced by the HIV Type 1-Envelope Glycoprotein Complex: Role of Mitochondria and Caspases. J. Exp. Med..

[55] Murooka T.T., Deruaz M., Marangoni F., Vrbanac V.D., Seung E., von Andrian U.H., Tager A.M., Luster A.D., Mempel T.R. (2012). HIV-Infected T Cells Are Migratory Vehicles for Viral Dissemination. Nature.

[56] Rashid F., Zaongo S.D., Iqbal H., Harypursat V., Song F., Chen Y. (2024). Interactions between HIV Proteins and Host Restriction Factors: Implications for Potential Therapeutic Intervention in HIV Infection. Front. Immunol..

[57] Kmiec D., Kirchhoff F. (2024). Antiviral Factors and Their Counteraction by HIV-1: Many Uncovered and More to Be Discovered. J. Mol. Cell Biol..

[58] Goff S.P. (2003). Death by Deamination: A Novel Host Restriction System for HIV-1. Cell.

[59] Zhang F., Bieniasz P.D. (2020). HIV-1 Vpr Induces Cell Cycle Arrest and Enhances Viral Gene Expression by Depleting CCDC137. eLife.

[60] Neil S.J.D., Zang T., Bieniasz P.D. (2008). Tetherin Inhibits Retrovirus Release and Is Antagonized by HIV-1 Vpu. Nature.

[61] Iwabu Y., Fujita H., Kinomoto M., Kaneko K., Ishizaka Y., Tanaka Y., Sata T., Tokunaga K. (2009). HIV-1 Accessory Protein Vpu Internalizes Cell-Surface BST-2/Tetherin through Transmembrane Interactions Leading to Lysosomes. J. Biol. Chem..

[62] Volcic M., Wiesmüller L., Kirchhoff F. (2023). Small but Highly Versatile: The Viral Accessory Protein Vpu. Annu. Rev. Virol..

[63] Shen Q.-T., Ren X., Zhang R., Lee I.-H., Hurley J.H. (2015). HIV-1 Nef Hijacks Clathrin Coats by Stabilizing AP-1:Arf1 Polygons. Science.

[64] Gondim M.V., Wiltzer-Bach L., Maurer B., Banning C., Arganaraz E., Schindler M. (2015). AP-2 Is the Crucial Clathrin Adaptor Protein for CD4 Downmodulation by HIV-1 Nef in Infected Primary CD4+ T Cells. J. Virol..

[65] Schwartz O., Maréchal V., Le Gall S., Lemonnier F., Heard J.M. (1996). Endocytosis of Major Histocompatibility Complex Class I Molecules Is Induced by the HIV-1 Nef Protein. Nat. Med..

[66] Fenard D., Yonemoto W., de Noronha C., Cavrois M., Williams S.A., Greene W.C. (2005). Nef Is Physically Recruited into the Immunological Synapse and Potentiates T Cell Activation Early after TCR Engagement. J. Immunol..

[67] Bekker L.-G., Beyrer C., Mgodi N., Lewin S.R., Delany-Moretlwe S., Taiwo B., Masters M.C., Lazarus J.V. (2023). HIV Infection. Nat. Rev. Dis. Prim..

[68] Alcamí J., Coiras M. (2011). Immunopathogenesis of HIV infection. Enfermedades Infecc. Microbiol. Clínica.

[69] Centlivre M., Sala M., Wain-Hobson S., Berkhout B. (2007). In HIV-1 Pathogenesis the Die Is Cast during Primary Infection. Aids.

[70] Sabin C.A., Lundgren J.D. (2013). The Natural History of HIV Infection. Curr. Opin. HIV AIDS.

[71] McMichael A.J., Borrow P., Tomaras G.D., Goonetilleke N., Haynes B.F. (2010). The Immune Response during Acute HIV-1 Infection: Clues for Vaccine Development. Nat. Rev. Immunol..

[72] Stacey A.R., Norris P.J., Qin L., Haygreen E.A., Taylor E., Heitman J., Lebedeva M., DeCamp A., Li D., Grove D. (2009). Induction of a Striking Systemic Cytokine Cascade Prior to Peak Viremia in Acute Human Immunodeficiency Virus Type 1 Infection, in Contrast to More Modest and Delayed Responses in Acute Hepatitis B and C Virus Infections. J. Virol..

[73] Gay C., Dibben O., Anderson J.A., Stacey A., Mayo A.J., Norris P.J., Kuruc J.D., Salazar-Gonzalez J.F., Li H., Keele B.F. (2011). Cross-Sectional Detection of Acute HIV Infection: Timing of Transmission, Inflammation and Antiretroviral Therapy. PLoS ONE.

[74] Pastor L., Urrea V., Carrillo J., Parker E., Fuente-Soro L., Jairoce C., Mandomando I., Naniche D., Blanco J. (2018). Dynamics of CD4 and CD8 T-Cell Subsets and Inflammatory Biomarkers during Early and Chronic HIV Infection in Mozambican Adults. Front. Immunol..

[75] Norris P.J., Pappalardo B.L., Custer B., Spotts G., Hecht F.M., Busch M.P. (2006). Elevations in IL-10, TNF-Alpha, and IFN-Gamma from the Earliest Point of HIV Type 1 Infection. AIDS Res. Hum. Retroviruses.

[76] Roberts L., Passmore J.-A.S.A., Williamson C., Little F., Bebell L.M., Mlisana K., Burgers W.A., van Loggerenberg F., Walzl G., Djoba Siawaya J.F. (2010). Plasma Cytokine Levels during Acute HIV-1 Infection Predict HIV Disease Progression. Aids.

[77] Kramer H.B., Lavender K.J., Qin L., Stacey A.R., Liu M.K.P., di Gleria K., Simmons A., Gasper-Smith N., Haynes B.F., McMichael A.J. (2010). Elevation of Intact and Proteolytic Fragments of Acute Phase Proteins Constitutes the Earliest Systemic Antiviral Response in HIV-1 Infection. PLoS Pathog..

[78] Pastor L. (2017). Identification of Immune Biomarkersfor Use in Early HIV Detection and Monitoring in Sub-Saharian Africa. Ph.D. Thesis.

[79] Borrow P., Bhardwaj N. (2008). Innate Immune Responses in Primary HIV-1 Infection. Curr. Opin. HIV AIDS.

[80] Ploquin M.J., Madec Y., Casrouge A., Huot N., Passaes C., Lécuroux C., Essat A., Boufassa F., Jacquelin B., Jochems S.P. (2016). Elevated Basal Pre-Infection CXCL10 in Plasma and in the Small Intestine after Infection Are Associated with More Rapid HIV/SIV Disease Onset. PLoS Pathog..

[81] Tomaras G.D., Haynes B.F. (2009). HIV-1-Specific Antibody Responses during Acute and Chronic HIV-1 Infection. Curr. Opin. HIV AIDS.

[82] Tomaras G.D., Yates N.L., Liu P., Qin L., Fouda G.G., Chavez L.L., Decamp A.C., Parks R.J., Ashley V.C., Lucas J.T. (2008). Initial B-Cell Responses to Transmitted Human Immunodeficiency Virus Type 1: Virion-Binding Immunoglobulin M (IgM) and IgG Antibodies Followed by Plasma Anti-Gp41 Antibodies with Ineffective Control of Initial Viremia. J. Virol..

[83] Wei X., Decker J.M., Wang S., Hui H., Kappes J.C., Wu X., Salazar-Gonzalez J.F., Salazar M.G., Kilby J.M., Saag M.S. (2003). Antibody Neutralization and Escape by HIV-1. Nature.

[84] Gray E.S., Moore P.L., Choge I.A., Decker J.M., Bibollet-Ruche F., Li H., Leseka N., Treurnicht F., Mlisana K., Shaw G.M. (2007). Neutralizing Antibody Responses in Acute Human Immunodeficiency Virus Type 1 Subtype C Infection. J. Virol..

[85] Richman D.D., Wrin T., Little S.J., Petropoulos C.J. (2003). Rapid Evolution of the Neutralizing Antibody Response to HIV Type 1 Infection. Proc. Natl. Acad. Sci. USA.

[86] Stamatatos L., Morris L., Burton D.R., Mascola J.R. (2009). Neutralizing Antibodies Generated during Natural HIV-1 Infection: Good News for an HIV-1 Vaccine?. Nat. Med..

[87] Gray E.S., Madiga M.C., Moore P.L., Mlisana K., Abdool Karim S.S., Binley J.M., Shaw G.M., Mascola J.R., Morris L. (2009). Broad Neutralization of Human Immunodeficiency Virus Type 1 Mediated by Plasma Antibodies against the Gp41 Membrane Proximal External Region. J. Virol..

[88] Rusert P., Kouyos R.D., Kadelka C., Ebner H., Schanz M., Huber M., Braun D.L., Hozé N., Scherrer A., Magnus C. (2016). Determinants of HIV-1 Broadly Neutralizing Antibody Induction. Nat. Med..

[89] Simek M.D., Rida W., Priddy F.H., Pung P., Carrow E., Laufer D.S., Lehrman J.K., Boaz M., Tarragona-Fiol T., Miiro G. (2009). Human Immunodeficiency Virus Type 1 Elite Neutralizers: Individuals with Broad and Potent Neutralizing Activity Identified by Using a High-Throughput Neutralization Assay Together with an Analytical Selection Algorithm. J. Virol..

[90] Ndhlovu Z.M., Kamya P., Mewalal N., Kløverpris H.N., Nkosi T., Pretorius K., Laher F., Ogunshola F., Chopera D., Shekhar K. (2015). Magnitude and Kinetics of CD8+ T Cell Activation during Hyperacute HIV Infection Impact Viral Set Point. Immunity.

[91] Day C.L., Kaufmann D.E., Kiepiela P., Brown J.A., Moodley E.S., Reddy S., Mackey E.W., Miller J.D., Leslie A.J., DePierres C. (2006). PD-1 Expression on HIV-Specific T Cells Is Associated with T-Cell Exhaustion and Disease Progression. Nature.

[92] Hoffmann M., Pantazis N., Martin G.E., Hickling S., Hurst J., Meyerowitz J., Willberg C.B., Robinson N., Brown H., Fisher M. (2016). Exhaustion of Activated CD8 T Cells Predicts Disease Progression in Primary HIV-1 Infection. PLoS Pathog..

[93] Robb M.L., Eller L.A., Kibuuka H., Rono K., Maganga L., Nitayaphan S., Kroon E., Sawe F.K., Sinei S., Sriplienchan S. (2016). Prospective Study of Acute HIV-1 Infection in Adults in East Africa and Thailand. N. Engl. J. Med..

[94] Liu R., Simonetti F.R., Ho Y.-C. (2020). The Forces Driving Clonal Expansion of the HIV-1 Latent Reservoir. Virol. J..

[95] Lefrançois L., Puddington L. (2006). Intestinal and Pulmonary Mucosal T Cells: Local Heroes Fight to Maintain the Status Quo. Annu. Rev. Immunol..

[96] Mattapallil J.J., Douek D.C., Hill B., Nishimura Y., Martin M., Roederer M. (2005). Massive Infection and Loss of Memory CD4+ T Cells in Multiple Tissues during Acute SIV Infection. Nature.

[97] Brenchley J.M., Schacker T.W., Ruff L.E., Price D.A., Taylor J.H., Beilman G.J., Nguyen P.L., Khoruts A., Larson M., Haase A.T. (2004). CD4+ T Cell Depletion during All Stages of HIV Disease Occurs Predominantly in the Gastrointestinal Tract. J. Exp. Med..

[98] Brenchley J.M., Price D.A., Schacker T.W., Asher T.E., Silvestri G., Rao S., Kazzaz Z., Bornstein E., Lambotte O., Altmann D. (2006). Microbial Translocation Is a Cause of Systemic Immune Activation in Chronic HIV Infection. Nat. Med..

[99] Rosás-Umbert M., Llano A., Bellido R., Olvera A., Ruiz-Riol M., Rocafort M., Fernández M.A., Cobarsi P., Crespo M., Dorrell L. (2019). Mechanisms of Abrupt Loss of Virus Control in a Cohort of Previous HIV Controllers. J. Virol..

[100] Soper A., Kimura I., Nagaoka S., Konno Y., Yamamoto K., Koyanagi Y., Sato K. (2018). Type I Interferon Responses by HIV-1 Infection: Association with Disease Progression and Control. Front. Immunol.

[101] Marchetti G., Gori A., Casabianca A., Magnani M., Franzetti F., Clerici M., Perno C.-F.F., Antonella d’Arminio M., Galli M., Meroni L. (2006). Comparative Analysis of T-Cell Turnover and Homeostatic Parameters in HIV-Infected Patients with Discordant Immune-Virological Responses to HAART. Aids.

[102] Hunt P.W., Martin J.N., Sinclair E., Epling L., Teague J., Jacobson M.A., Tracy R.P., Corey L., Deeks S.G. (2011). Valganciclovir Reduces T Cell Activation in HIV-Infected Individuals with Incomplete CD4+ T Cell Recovery on Antiretroviral Therapy. J. Infect. Dis..

[103] Deeks S.G. (2011). HIV Infection, Inflammation, Immunosenescence, and Aging. Annu. Rev. Med..

[104] Jia F., Brew B.J. (2025). Neuropathogenesis of Acute HIV: Mechanisms, Biomarkers, and Therapeutic Approaches. Curr. Opin. HIV AIDS.

[105] Wallace D.R. (2022). HIV-Associated Neurotoxicity and Cognitive Decline: Therapeutic Implications. Pharmacol. Ther..

[106] Vieira V., Lim N., Singh A., Leitman E., Dsouza R., Adland E., Muenchhoff M., Roider J., Marin Lopez M., Carabelli J. (2023). Slow Progression of Pediatric HIV Associates with Early CD8+ T Cell PD-1 Expression and a Stem-like Phenotype. JCI Insight.

[107] Gurdasani D., Iles L., Dillon D.G., Young E.H., Olson A.D., Naranbhai V., Fidler S., Gkrania-Klotsas E., Post F.A., Kellam P. (2014). A Systematic Review of Definitions of Extreme Phenotypes of HIV Control and Progression. Aids.

[108] Velasco C., Parker E., Pastor L., Nhama A., Macuacua S., Mandomando I., Blanco J., Naniche D. (2015). Case Report: Rapid HIV Progression during Acute HIV-1 Subtype C Infection in a Mozambican Patient with Atypical Seroconversion. Am. J. Trop. Med. Hyg..

[109] Spivak A.M., Sydnor E.R.M., Blankson J.N., Gallant J.E. (2010). Seronegative HIV-1 Infection: A Review of the Literature. Aids.

[110] Sundaramurthi J.C., Ashokkumar M., Swaminathan S., Hanna L.E. (2017). HLA Based Selection of Epitopes Offers a Potential Window of Opportunity for Vaccine Design against HIV. Vaccine.

[111] Payne R.P., Kløverpris H., Sacha J.B., Brumme Z., Brumme C., Buus S., Sims S., Hickling S., Riddell L., Chen F. (2010). Efficacious Early Antiviral Activity of HIV Gag- and Pol-Specific HLA-B 2705-Restricted CD8+ T Cells. J. Virol..

[112] Oriol-Tordera B., Llano A., Ganoza C., Cate S., Hildebrand W., Sanchez J., Calle M.L., Brander C., Olvera A. (2017). Impact of HLA-DRB1 Allele Polymorphisms on Control of HIV Infection in a Peruvian MSM Cohort. HLA.

[113] Pernas M., Tarancón-Diez L., Rodríguez-Gallego E., Gómez J., Prado J.G., Casado C., Dominguez-Molina B., Olivares I., Coiras M., León A. (2018). Factors Leading to the Loss of Natural Elite Control of HIV-1 Infection. J. Virol..

[114] Moore J.P., Kitchen S.G., Pugach P., Zack J.A. (2004). The CCR5 and CXCR4 Coreceptors—Central to Understanding the Transmission and Pathogenesis of Human Immunodeficiency Virus Type 1 Infection. AIDS Res. Hum. Retroviruses.

[115] Kalidasan V., Theva Das K. (2020). Lessons Learned From Failures and Success Stories of HIV Breakthroughs: Are We Getting Closer to an HIV Cure?. Front. Microbiol..

[116] Zaunders J.J., Geczy A.F., Dyer W.B., McIntyre L.B., Cooley M.A., Ashton L.J., Raynes-Greenow C.H., Learmont J., Cooper D.A., Sullivan J.S. (1999). Effect of Long-Term Infection with Nef-Defective Attenuated HIV Type 1 on CD4+ and CD8+ T Lymphocytes: Increased CD45RO+CD4+ T Lymphocytes and Limited Activation of CD8+ T Lymphocytes. AIDS Res. Hum. Retroviruses.

[117] Lassen K.G., Lobritz M.A., Bailey J.R., Johnston S., Nguyen S., Lee B., Chou T., Siliciano R.F., Markowitz M., Arts E.J. (2009). Elite Suppressor-Derived HIV-1 Envelope Glycoproteins Exhibit Reduced Entry Efficiency and Kinetics. PLoS Pathog..

[118] Pérez-Yanes S., Pernas M., Marfil S., Cabrera-Rodríguez R., Ortiz R., Urrea V., Rovirosa C., Estévez-Herrera J., Olivares I., Casado C. (2022). The Characteristics of the HIV-1 Env Glycoprotein Are Linked with Viral Pathogenesis. Front. Microbiol..

[119] Casado C., Marrero-Hernández S., Márquez-Arce D., Pernas M., Marfil S., Borràs-Grañana F., Olivares I., Cabrera-Rodríguez R., Valera M.-S.S., de Armas-Rillo L. (2018). Viral Characteristics Associated with the Clinical Nonprogressor Phenotype Are Inherited by Viruses from a Cluster of HIV-1 Elite Controllers. mBio.

[120] Lopez-Galindez C., Pernas M., Casado C., Olivares I., Lorenzo-Redondo R. (2019). Elite Controllers and Lessons Learned for HIV-1 Cure. Curr. Opin. Virol..

[121] Fiebig E.W., Wright D.J., Rawal B.D., Garrett P.E., Schumacher R.T., Peddada L., Heldebrant C., Smith R., Conrad A., Kleinman S.H. (2003). Dynamics of HIV Viremia and Antibody Seroconversion in Plasma Donors: Implications for Diagnosis and Staging of Primary HIV Infection. AIDS.

[122] Gökengin D., Wilson-Davies E., Nazlı Zeka A., Palfreeman A., Begovac J., Dedes N., Tarashenko O., Stevanovic M., Patel R. (2021). 2021 European Guideline on HIV Testing in Genito-Urinary Medicine Settings. J. Eur. Acad. Dermatol. Venereol..

[123] Witzel T.C., Eshun-Wilson I., Jamil M.S., Tilouche N., Figueroa C., Johnson C.C., Reid D., Baggaley R., Siegfried N., Burns F.M. (2020). Comparing the Effects of HIV Self-Testing to Standard HIV Testing for Key Populations: A Systematic Review and Meta-Analysis. BMC Med..

[124] Serrano-Villar S., Wu K., Hunt P.W., Lok J.J., Ron R., Sainz T., Moreno S., Deeks S.G., Bosch R.J. (2022). Predictive Value of CD8+ T Cell and CD4/CD8 Ratio at Two Years of Successful ART in the Risk of AIDS and Non-AIDS Events. eBioMedicine.

[125] Fischl M.A., Richman D.D., Grieco M.H., Gottlieb M.S., Volberding P.A., Laskin O.L., Leedom J.M., Groopman J.E., Mildvan D., Schooley R.T. (1987). The Efficacy of Azidothymidine (AZT) in the Treatment of Patients with AIDS and AIDS-Related Complex. A Double-Blind, Placebo-Controlled Trial. N. Engl. J. Med..

[126] Fischl M.A. (1994). Combination Antiretroviral Therapy for HIV Infection. Hosp. Pract..

[127] Pau A.K., George J.M. (2014). Antiretroviral Therapy: Current Drugs. Infect. Dis. Clin. N. Am..

[128] Hammer S.M., Squires K.E., Hughes M.D., Grimes J.M., Demeter L.M., Currier J.S., Eron J.J.J., Feinberg J.E., Balfour H.H.J., Deyton L.R. (1997). A Controlled Trial of Two Nucleoside Analogues plus Indinavir in Persons with Human Immunodeficiency Virus Infection and CD4 Cell Counts of 200 per Cubic Millimeter or Less. AIDS Clinical Trials Group 320 Study Team. N. Engl. J. Med..

[129] Kemnic T.R., Gulick P.G. (2021). HIV Antiretroviral Therapy.

[130] Neverette N.C., Dumond J.B., McMahon D.K., Devanathan A.S. (2024). Lenacapavir: Playing the Long Game in the New Era of Antiretrovirals. Clin. Pharmacol. Ther..

[131] Xu F., Acosta E.P., Liang L., He Y., Yang J., Kerstner-Wood C., Zheng Q., Huang J., Wang K. (2017). Current Status of the Pharmacokinetics and Pharmacodynamics of HIV-1 Entry Inhibitors and HIV Therapy. Curr. Drug Metab..

[132] Saag M.S., Gandhi R.T., Hoy J.F., Landovitz R.J., Thompson M.A., Sax P.E., Smith D.M., Benson C.A., Buchbinder S.P., Del Rio C. (2020). Antiretroviral Drugs for Treatment and Prevention of HIV Infection in Adults: 2020 Recommendations of the International Antiviral Society-USA Panel. JAMA.

[133] Grasberger P., Clayton K.L. (2025). Targeting HIV Myeloid and Central Nervous System Reservoirs for HIV Cure. Curr. Opin. HIV AIDS.

[134] Plana M., García F., Gallart T., Tortajada C., Soriano A., Palou E., Maleno M.J., Barceló J.J., Vidal C., Cruceta A. (2000). Immunological Benefits of Antiretroviral Therapy in Very Early Stages of Asymptomatic Chronic HIV-1 Infection. AIDS.

[135] Riddell IV J., Amico K.R., Mayer K.H. (2018). HIV Preexposure Prophylaxis: A Review. JAMA.

[136] Landovitz R.J., Donnell D., Clement M.E., Hanscom B., Cottle L., Coelho L., Cabello R., Chariyalertsak S., Dunne E.F., Frank I. (2021). Cabotegravir for HIV Prevention in Cisgender Men and Transgender Women. N. Engl. J. Med..

[137] Kelley C.F., Acevedo-Quiñones M., Agwu A.L., Avihingsanon A., Benson P., Blumenthal J., Brinson C., Brites C., Cahn P., Cantos V.D. (2024). Twice-Yearly Lenacapavir for HIV Prevention in Men and Gender-Diverse Persons. N. Engl. J. Med..

[138] Carrillo J., Clotet B., Blanco J. (2018). Antibodies and Antibody Derivatives: New Partners in HIV Eradication Strategies. Front. Immunol..

[139] Puertas M.C., Massanella M., Llibre J.M., Ballestero M., Buzon M.J., Ouchi D., Esteve A., Boix J., Manzardo C., Miró J.M. (2014). Intensification of a Raltegravir-Based Regimen with Maraviroc in Early HIV-1 Infection. AIDS.

[140] Pierson T., McArthur J., Siliciano R.F. (2000). Reservoirs for HIV-1: Mechanisms for Viral Persistence in the Presence of Antiviral Immune Responses and Antiretroviral Therapy. Annu. Rev. Immunol..

[141] Haworth K.G., Schefter L.E., Norgaard Z.K., Ironside C., Adair J.E., Kiem H.-P.P. (2018). HIV Infection Results in Clonal Expansions Containing Integrations within Pathogenesis-Related Biological Pathways. JCI Insight.

[142] Pakker N.G., Notermans D.W., de Boer R.J., Roos M.T., de Wolf F., Hill A., Leonard J.M., Danner S.A., Miedema F., Schellekens P.T. (1998). Biphasic Kinetics of Peripheral Blood T Cells after Triple Combination Therapy in HIV-1 Infection: A Composite of Redistribution and Proliferation. Nat. Med..

[143] Massanella M., Ouchi D., Marfil S., Llibre J.M., Puertas M.C., Buzón M.J., Richman D.D., Orna E., Stevenson M., Gatell J.M. (2014). Different Plasma Markers of Inflammation Are Influenced by Immune Recovery and CART Composition or Intensification in Treated HIV Infected Individuals. PLoS ONE.

[144] Negredo E., Back D., Blanco J.-R.R.J.J.R., Blanco J.-R.R.J.J.R., Erlandson K.M., Garolera M., Guaraldi G., Mallon P., Moltó J., Serra J.A. (2017). Aging in HIV-Infected Subjects: A New Scenario and a New View. BioMed Res. Int..

[145] Massanella M., Negredo E., Clotet B., Blanco J. (2013). Immunodiscordant Responses to HAART-Mechanisms and Consequences. Expert Rev. Clin. Immunol..

[146] Corbeau P., Reynes J. (2011). Immune Reconstitution under Antiretroviral Therapy: The New Challenge in HIV-1 Infection. Blood.

[147] Massanella M., Negredo E., Pérez-Álvarez N., Puig J., Ruiz-Hernández R., Bofill M., Clotet B., Blanco J., Pérez-Alvarez N., Puig J. (2010). CD4 T-Cell Hyperactivation and Susceptibility to Cell Death Determine Poor CD4 T-Cell Recovery during Suppressive HAART. AIDS.

[148] Pacheco Y.M., Jarrin I., Rosado I., Campins A.A., Berenguer J., Iribarren J.A., Rivero M., Muñoz-Medina L., Bernal-Morell E., Gutiérrez F. (2015). Increased Risk of Non-AIDS-Related Events in HIV Subjects with Persistent Low CD4 Counts despite CART in the CoRIS Cohort. Antivir. Res..

[149] Bonsignori M., Liao H.-X., Gao F., Williams W.B., Alam S.M., Montefiori D.C., Haynes B.F. (2017). Antibody-Virus Co-Evolution in HIV Infection: Paths for HIV Vaccine Development. Immunol. Rev..

[150] Sadanand S., Suscovich T.J., Alter G. (2016). Broadly Neutralizing Antibodies Against HIV: New Insights to Inform Vaccine Design. Annu. Rev. Med..

[151] Wang Q., Zhang L. (2020). Broadly Neutralizing Antibodies and Vaccine Design against HIV-1 Infection. Front. Med..

[152] Klein F., Diskin R., Scheid J.F., Gaebler C., Mouquet H., Georgiev I.S., Pancera M., Zhou T., Incesu R.-B., Fu B.Z. (2013). Somatic Mutations of the Immunoglobulin Framework Are Generally Required for Broad and Potent HIV-1 Neutralization. Cell.

[153] Breden F., Lepik C., Longo N.S., Montero M., Lipsky P.E., Scott J.K. (2011). Comparison of Antibody Repertoires Produced by HIV-1 Infection, Other Chronic and Acute Infections, and Systemic Autoimmune Disease. PLoS ONE.

[154] Euler Z., van Gils M.J., Bunnik E.M., Phung P., Schweighardt B., Wrin T., Schuitemaker H. (2010). Cross-Reactive Neutralizing Humoral Immunity Does Not Protect from HIV Type 1 Disease Progression. J. Infect. Dis..

[155] Gauduin M.C., Parren P.W., Weir R., Barbas C.F., Burton D.R., Koup R.A. (1997). Passive Immunization with a Human Monoclonal Antibody Protects Hu-PBL-SCID Mice against Challenge by Primary Isolates of HIV-1. Nat. Med..

[156] Shibata R., Igarashi T., Haigwood N., Buckler-White A., Ogert R., Ross W., Willey R., Cho M.W., Martin M.A. (1999). Neutralizing Antibody Directed against the HIV-1 Envelope Glycoprotein Can Completely Block HIV-1/SIV Chimeric Virus Infections of Macaque Monkeys. Nat. Med..

[157] Mascola J.R. (2002). Passive Transfer Studies to Elucidate the Role of Antibody-Mediated Protection against HIV-1. Vaccine.

[158] Corey L., Gilbert P.B., Juraska M., Montefiori D.C., Morris L., Karuna S.T., Edupuganti S., Mgodi N.M., deCamp A.C., Rudnicki E. (2021). Two Randomized Trials of Neutralizing Antibodies to Prevent HIV-1 Acquisition. N. Engl. J. Med..

[159] Ananworanich J., McSteen B., Robb M.L. (2015). Broadly Neutralizing Antibody and the HIV Reservoir in Acute HIV Infection: A Strategy toward HIV Remission?. Curr. Opin. HIV AIDS.

[160] Rerks-Ngarm S., Brown A.E., Khamboonruang C., Thongcharoen P., Kunasol P. (2006). HIV/AIDS Preventive Vaccine ‘Prime-Boost’ Phase III Trial: Foundations and Initial Lessons Learned from Thailand. AIDS.

[161] Burton D.R., Desrosiers R.C., Doms R.W., Feinberg M.B., Gallo R.C., Hahn B., Hoxie J.A., Hunter E., Korber B., Landay A. (2004). A Sound Rationale Needed for Phase III HIV-1 Vaccine Trials. Science.

[162] Haynes B.F., Gilbert P.B., McElrath M.J., Zolla-Pazner S., Tomaras G.D., Alam S.M., Evans D.T., Montefiori D.C., Karnasuta C., Sutthent R. (2012). Immune-Correlates Analysis of an HIV-1 Vaccine Efficacy Trial. N. Engl. J. Med..

[163] Wren L., Kent S.J. (2011). HIV Vaccine Efficacy Trial: Glimmers of Hope and the Potential Role of Antibody-Dependent Cellular Cytotoxicity. Hum. Vaccines.

[164] Mothe B., Hu X., Llano A., Rosati M., Olvera A., Kulkarni V., Valentin A., Alicea C., Pilkington G.R., Sardesai N.Y. (2015). A Human Immune Data-Informed Vaccine Concept Elicits Strong and Broad T-Cell Specificities Associated with HIV-1 Control in Mice and Macaques. J. Transl. Med..

[165] Fischer W., Perkins S., Theiler J., Bhattacharya T., Yusim K., Funkhouser R., Kuiken C., Haynes B., Letvin N.L., Walker B.D. (2007). Polyvalent Vaccines for Optimal Coverage of Potential T-Cell Epitopes in Global HIV-1 Variants. Nat. Med..

[166] Barouch D.H., O’Brien K.L., Simmons N.L., King S.L., Abbink P., Maxfield L.F., Sun Y.-H., La Porte A., Riggs A.M., Lynch D.M. (2010). Mosaic HIV-1 Vaccines Expand the Breadth and Depth of Cellular Immune Responses in Rhesus Monkeys. Nat. Med..

[167] Gilbert P.B., Peterson M.L., Follmann D., Hudgens M.G., Francis D.P., Gurwith M., Heyward W.L., Jobes D.V., Popovic V., Self S.G. (2005). Correlation between Immunologic Responses to a Recombinant Glycoprotein 120 Vaccine and Incidence of HIV-1 Infection in a Phase 3 HIV-1 Preventive Vaccine Trial. J. Infect. Dis..

[168] Gallo R.C. (2005). The End or the Beginning of the Drive to an HIV-Preventive Vaccine: A View from over 20 Years. Lancet.

[169] Pitisuttithum P., Gilbert P., Gurwith M., Heyward W., Martin M., van Griensven F., Hu D., Tappero J.W., Choopanya K., Group B.V.E. (2006). Randomized, Double-Blind, Placebo-Controlled Efficacy Trial of a Bivalent Recombinant Glycoprotein 120 HIV-1 Vaccine among Injection Drug Users in Bangkok, Thailand. J. Infect. Dis..

[170] Flynn N.M., Forthal D.N., Harro C.D., Judson F.N., Mayer K.H., Para M.F. (2005). Placebo-Controlled Phase 3 Trial of a Recombinant Glycoprotein 120 Vaccine to Prevent HIV-1 Infection. J. Infect. Dis..

[171] Klasse P.J., Ozorowski G., Sanders R.W., Moore J.P. (2020). Env Exceptionalism: Why Are HIV-1 Env Glycoproteins Atypical Immunogens?. Cell Host Microbe.

[172] Thalhauser S., Peterhoff D., Wagner R., Breunig M. (2020). Critical Design Criteria for Engineering a Nanoparticulate HIV-1 Vaccine. J. Control. Release.

[173] Sanders R.W., Vesanen M., Schuelke N., Master A., Schiffner L., Kalyanaraman R., Paluch M., Berkhout B., Maddon P.J., Olson W.C. (2002). Stabilization of the Soluble, Cleaved, Trimeric Form of the Envelope Glycoprotein Complex of Human Immunodeficiency Virus Type 1. J. Virol..

[174] Julien J.-P., Lee J.H., Ozorowski G., Hua Y., Torrents de la Peña A., de Taeye S.W., Nieusma T., Cupo A., Yasmeen A., Golabek M. (2015). Design and Structure of Two HIV-1 Clade C SOSIP.664 Trimers That Increase the Arsenal of Native-like Env Immunogens. Proc. Natl. Acad. Sci. USA.

[175] Sliepen K., Han B.W., Bontjer I., Mooij P., Garces F., Behrens A.-J., Rantalainen K., Kumar S., Sarkar A., Brouwer P.J.M. (2019). Structure and Immunogenicity of a Stabilized HIV-1 Envelope Trimer Based on a Group-M Consensus Sequence. Nat. Commun..

[176] Whitaker N., Hickey J.M., Kaur K., Xiong J., Sawant N., Cupo A., Lee W.-H., Ozorowski G., Medina-Ramírez M., Ward A.B. (2019). Developability Assessment of Physicochemical Properties and Stability Profiles of HIV-1 BG505 SOSIP.664 and BG505 SOSIP.v4.1-GT1.1 Gp140 Envelope Glycoprotein Trimers as Candidate Vaccine Antigens. J. Pharm. Sci..

[177] Tarrés-Freixas F., Clotet B., Carrillo J., Blanco J. (2024). Nucleic Acid Vaccines Encoding Proteins and Virus-like Particles for HIV Prevention. Vaccines.

[178] Tarrés-Freixas F., Aguilar-Gurrieri C., de la Concepción M.L.R., Urrea V., Trinité B., Ortiz R., Pradenas E., Blanco P., Marfil S., Molinos-Albert L.M. (2023). An Engineered HIV-1 Gag-Based VLP Displaying High Antigen Density Induces Strong Antibody-Dependent Functional Immune Responses. npj Vaccines.

[179] Sliepen K., Ozorowski G., Burger J.A., van Montfort T., Stunnenberg M., LaBranche C., Montefiori D.C., Moore J.P., Ward A.B., Sanders R.W. (2015). Presenting Native-like HIV-1 Envelope Trimers on Ferritin Nanoparticles Improves Their Immunogenicity. Retrovirology.

[180] Excler J.-L., Kim J.H. (2019). Novel Prime-Boost Vaccine Strategies against HIV-1. Expert Rev. Vaccines.

[181] Burton D.R. (2019). Advancing an HIV Vaccine; Advancing Vaccinology. Nat. Rev. Immunol..

[182] Jardine J.G., Kulp D.W., Havenar-Daughton C., Sarkar A., Briney B., Sok D., Sesterhenn F., Ereño-Orbea J., Kalyuzhniy O., Deresa I. (2016). HIV-1 Broadly Neutralizing Antibody Precursor B Cells Revealed by Germline-Targeting Immunogen. Science.

[183] Molinos-Albert L.M., Clotet B., Blanco J., Carrillo J. (2017). Immunologic Insights on the Membrane Proximal External Region: A Major Human Immunodeficiency Virus Type-1 Vaccine Target. Front. Immunol..

[184] Joint United Nations Programme on HIV/AIDS (2024). UNAIDS 2024.

[185] The World Bank Life Expectancy at Birth, Total (Years). https://data360.worldbank.org/en/indicator/WB_WDI_SP_DYN_LE00_IN?view=trend&country=USA&ave.

[186] Higgins J.A., Hoffman S., Dworkin S.L. (2010). Rethinking Gender, Heterosexual Men, and Women’s Vulnerability to HIV/AIDS. Am. J. Public Health.

